# Insights into the Ecotoxicity of Silver Nanoparticles Transferred from *Escherichia coli* to *Caenorhabditis elegans*

**DOI:** 10.1038/srep36465

**Published:** 2016-11-04

**Authors:** Xun Luo, Shengmin Xu, Yaning Yang, Luzhi Li, Shaopeng Chen, An Xu, Lijun Wu

**Affiliations:** 1 Key Laboratory of Ion Beam Bioengineering, Hefei Institutes of Physical Science, Chinese Academy of Sciences, Hefei, Anhui 230031, China; 2 School of Life Sciences, University of Science and Technology of China, Hefei 230026, Anhui, China; 3 School of Bioengineering, Huainan Normal University, Huainan 232038, China; 4 Key Laboratory of Environmental Toxicology and Pollution Control Technology of Anhui Province, Hefei, Anhui 230031, China

## Abstract

Previous studies have indicated that engineered nanomaterials can be transferred through the food chain. However, their potential ecotoxicity to the environment is not fully understood. Here, we systematically evaluated the physiological behavior and toxicity of polyvinylpyrrolidone (PVP)-coated silver nanoparticles (AgNPs) using a food chain model from *Escherichia coli* (*E. coli*) to *Caenorhabditis elegans* (*C. elegans*). Our results demonstrated that AgNPs accumulated in *E. coli* could be transferred to the *C. elegans*, and AgNPs were clearly distributed in the gut lumen, subcutaneous tissue and gonad. After being transferred to *C. elegans* through the food chain, the accumulated AgNPs caused serious toxicity to the higher trophic level (*C. elegans*), including effects on germ cell death, reproductive integrity and life span. Relative to larger particles (75 nm), small AgNPs (25 nm) more easily accumulated in the food chain and exhibited a stronger toxicity to the higher trophic level. More importantly, both the AgNPs that had accumulated in *C. elegans* through the food chain and the resulting impairment of germ cells could be transferred to the next generation, indicating that AgNP can cause genetic damage across generations. Our findings highlight that nanomaterials pose potential ecotoxicity to ecosystems via transport through the food chain.

Silver nanoparticles (AgNPs) and related products have been widely used in medicine and in commercialized products for their antimicrobial property^1^,^2^. Owing to the large-scale production and application of AgNP-related products, however, AgNPs will inevitably enter the environment^3^,^4^, and pose potential risks to the environment as well as to human health^5^,^6^. Many studies have demonstrated the toxic effects of AgNPs in a variety of models, such as in bacteria, mammalian cells and mice^7^,^8^,^9^. In a related aspect, the potential ecotoxicity of nanomaterials has become a principal object of research in recent years. For example, Roh *et al*. have investigated the ecotoxicity of AgNPs on the soil nematode *Caenorhabditis elegans* using functional ecotoxicogenomics^10^; by comparing the ecotoxicity of bare and coated AgNPs in the aquatic midge *Chironomus riparius*^11^, they found that the surface coating generally mitigated the toxicity of the nanoparticles. Meanwhile, Hund *et al*. obtained ecotoxicological data for AgNPs with the modified OECD test guidelines^12^.

Food chain transport is an important consideration for evaluations of the ecological toxicity of engineered nanomaterials (ENMs). There is evidence that ENMs can be transferred between trophic levels of different food chains, such as those of bacteria-protozoa^13^, bacteria-ciliate-rotifer^14^, and plant-worm^15^, and their distribution in predators has been confirmed using various methods. In addition, the trophic transfer of quantum dots has been demonstrated in a simplified invertebrate food web^13^,^14^. Another study reported the trophic transfer and biomagnification of gold nanoparticles from tobacco plants to tobacco hornworms^15^. These results demonstrate the potential trophic transfer of ENMs and raise the likelihood of human exposure through dietary uptake. Moreover, a few studies have focused on the toxicity of ENMs in food webs. For example, McTeer *et al*. assessed the toxicity and transfer of AgNPs in two model food chain organisms, from green *algae* to grazing *Daphnia*^16^. Chen *et al*. reported that nano-TiO_2_ was mildly toxic to *Scenedemus obliquus*, and could be transferred along the aquatic food chain with a biomagnification effect^17^. Chae *et al*. suggested the potential toxicological effects of silver nanowires in an aquatic food chain^18^. However, the behavior and toxicity of ENMs through food chains is not fully understood, especially the effects of specific nanomaterial properties, or their effects on subsequent generations.

*C. elegans* is a broadly distributed nematode species in soil ecosystems and plays a key role in nutrient cycling. Importantly, it has been widely employed as a test model in toxicology studies from the whole-animal level down to the single-cell level^10^,^19^. Moreover, *C. elegans* has a translucent body that can be exploited to observe the distribution of AgNPs in the organ system. Therefore, it is an excellent food chain model to elucidate the potential ecotoxicological effects of AgNPs. Here, we systematically studied the ecotoxicity of AgNPs by following their transfer from *Escherichia coli* to *C. elegans*. We found that a significant number of AgNPs that had accumulated in *E. coli* could be transferred to organisms at a higher trophic level (*C. elegans*). Moreover, the AgNP-treated *E. coli* cells were notably toxic to *C. elegans* in that they disturbed germ cell death and decreased the brood size and lifespan of the nematode. Importantly, we demonstrated that AgNPs accumulated in *C. elegans* through the food chain and that the impairment of germ cells could be transferred to the next generation. Our findings illustrate the potential ecotoxicity of AgNPs that are released into the environment onto food webs.

## Results

### Characterization of the AgNPs

**Figure 1:**
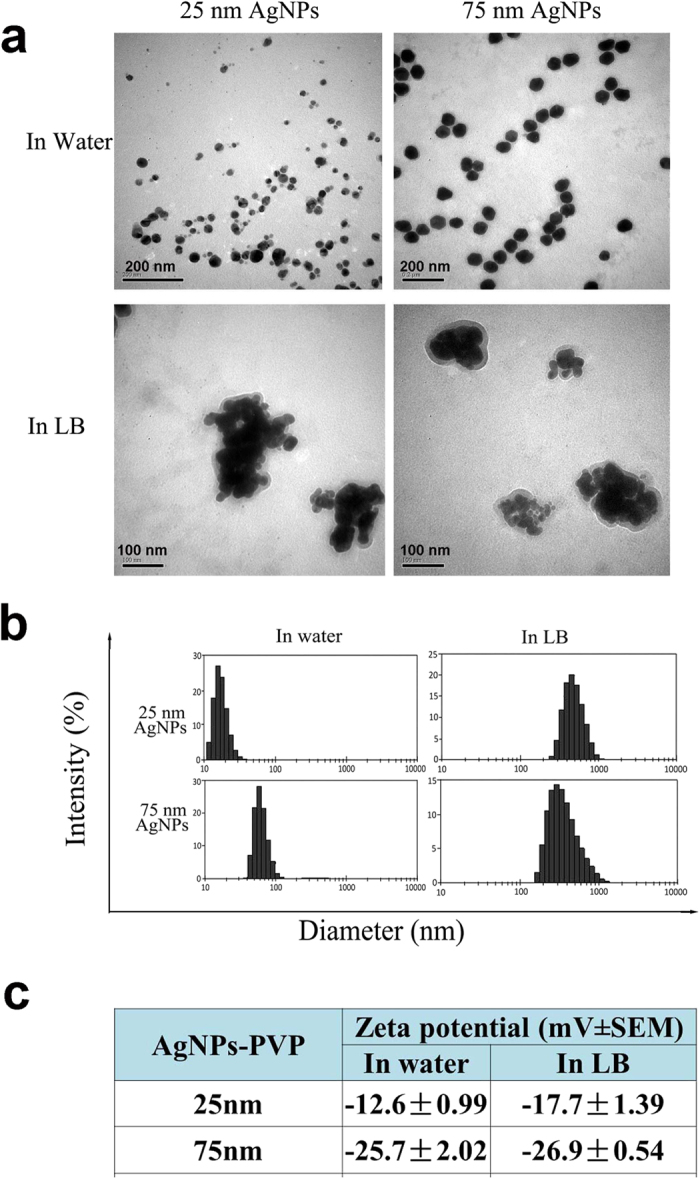
Characterization of AgNPs in water or LB medium. (**a**) Transmission electron microscopy (TEM) images of dispersed AgNPs in water or cultured in LB for 12 h. Scale bars equal 200 nm in water and 100 nm in LB. (**b**) The size distribution of AgNPs dispersed in water or LB medium as determined using dynamic light scattering (DLS). (**c**) Zeta potential of AgNPs in water or LB medium.

The two different-sized AgNPs (25 nm and 75 nm) used in our studies were first characterized using transmission electron microscopy (TEM), and hydrodynamic diameter and zeta potential analyses, under normal and test conditions. The AgNPs were monodispersed in distilled water, with a narrow size distribution (Fig. 1a). However, after incubation in the test medium (Luria Bertani, LB) for 12 h, TEM images showed that the AgNPs had aggregated greatly and that the hydrodynamic diameter had changed to 100–1000 nm. Although AgNP aggregation was extensive in the test medium, there were still some individual nano-sized particles in solution. The AgNP zeta potential was negative in both water and the medium, indicating that the nanoparticles could resist aggregation to some extent (Fig. 1c).

### Induction of mutigenerational *C. elegans* germ cell death by AgNPs through the food chain

**Figure 2:**
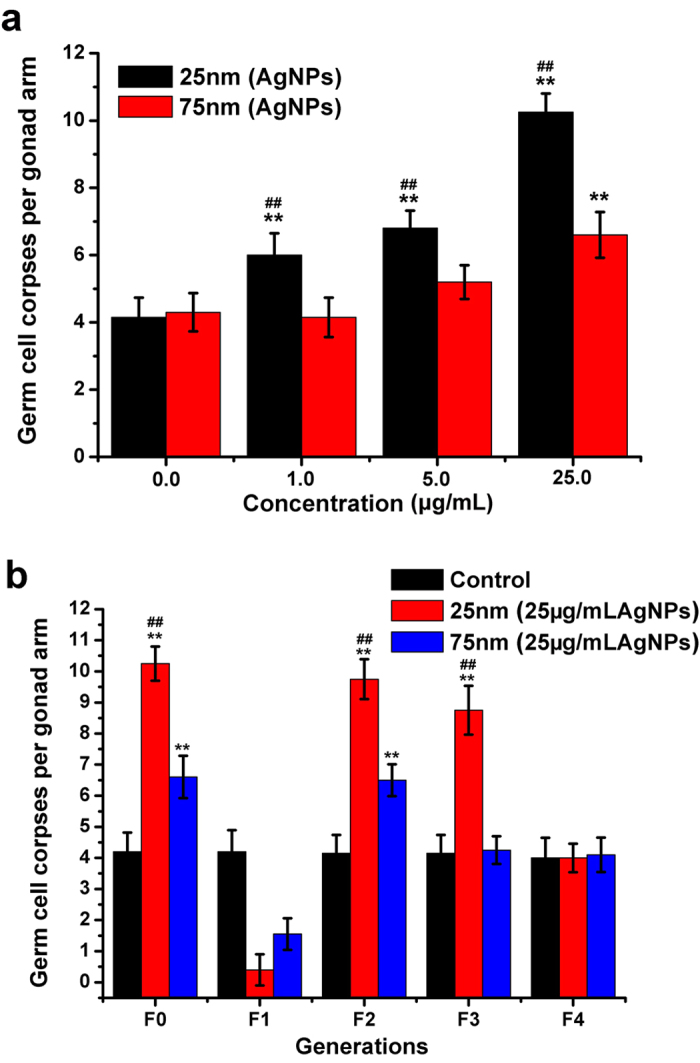
AgNPs induced germ cell death in *C. elegans* for all generations through the food chain. (**a**) Germ cell death in parental *C. elegans* induced by 25 nm and 75 nm AgNPs at concentrations of 1, 5 and 25 μg/mL through the food chain for 24 h. (**b**) Germ cell death in parental *C. elegans* induced by two size AgNPs at 25 μg/mL through the food chain transferred to subsequent generations (F1–F3). Only F0 was fed AgNPs-treated *E. coli* for 24 h. Subsequently, that F0 was moved to AgNPs-free NGM with fresh *E. coli* and allowed to reproduce next generations (F1 to F4). For the germ cell death assay in each generation, more than 20 *C. elegans* were screened in more than three independent experiments. The error bars indicate SD. Significant difference (**p < 0.01) from control, significant difference (^##^*p* < 0.01) between 25 nm and 75 nm AgNPs-treated group.

The toxicity of AgNPs to *C. elegans* through the food chain was first evaluated using a germ cell death assay, which represents an excellent end-point for toxicity assessment^20^,^21^. We first measured the impact of AgNPs on *E. coli*. As shown in Fig. S1a, *E. coli* growth was not significantly inhibited by either size of AgNPs at the concentrations of 1 and 5 μg/mL, but at the higher concentration of 25 μg/mL, the 25 nm nanoparticles could inhibit bacterial cell growth for up to 6 h post-treatment. However, all AgNP-treated *E. coli* cells reached the same stationary growth after 12 h. There was no significant reduction of colony number in response to AgNP exposure for 12 h (Fig. S1b). We further investigated the toxicity of AgNPs and Ag^+^ ions on *E. coli* using the flow cytometric method, which indicated that the AgNPs-treated *E. coli* cells used in our trophic transfer studies were mostly alive at the time of feeding to *C. elegans* (Fig. S1c). When the *C. elegans* worms were fed AgNP-treated *E. coli*, germ cell death of the nematode increased significantly in a dose-dependent manner. The 25 nm AgNPs were more toxic than the 75 nm nanoparticles (Fig. 2a), inducing significant germ cell death even at the lowest dose (1 μg/mL). Relative to the 25 nm AgNPs, the 75 nm nanoparticles did not induce obvious germ cell death until an exposure dose of up to 25 μg/mL. These results confirm that AgNPs could induce *C. elegans* germ cell death, and the smaller AgNPs were more toxic to germ cells than the larger nanoparticles.

The parental generation fed AgNPs-treated *E. coli* showed significant differences in germ cell death compared with the control group. We also performed a post-generation assay to study the toxic effects on subsequent generations that were incubated under AgNPs-free conditions. As shown in Fig. 2b, after removal of the AgNPs from the food chain, we confirmed that damaged F0 generations could transfer these adverse effects to subsequent generations. The gonads of the F1 generation were seriously impaired, and the germ cell death of generations F2 and F3 were higher than that in the control worms. The germ cell death receded gradually up to the F4 generation in the 25 nm AgNPs-treated group, whereas the recovery of germ cell death to normal levels was confirmed in the F3 generation in the 75 nm AgNPs-treated group. The transgenerational effects of the AgNPs may result from the impairment of germ cells caused by the nanoparticles to the parental generation. These results suggest that AgNPs in the food chain are potentially toxic to subsequent generations.

### AgNPs induction of germ cell death in *C. elegans* through the food chain is dependent on the accumulation and exclusion of AgNPs by *E. coli*

**Figure 3:**
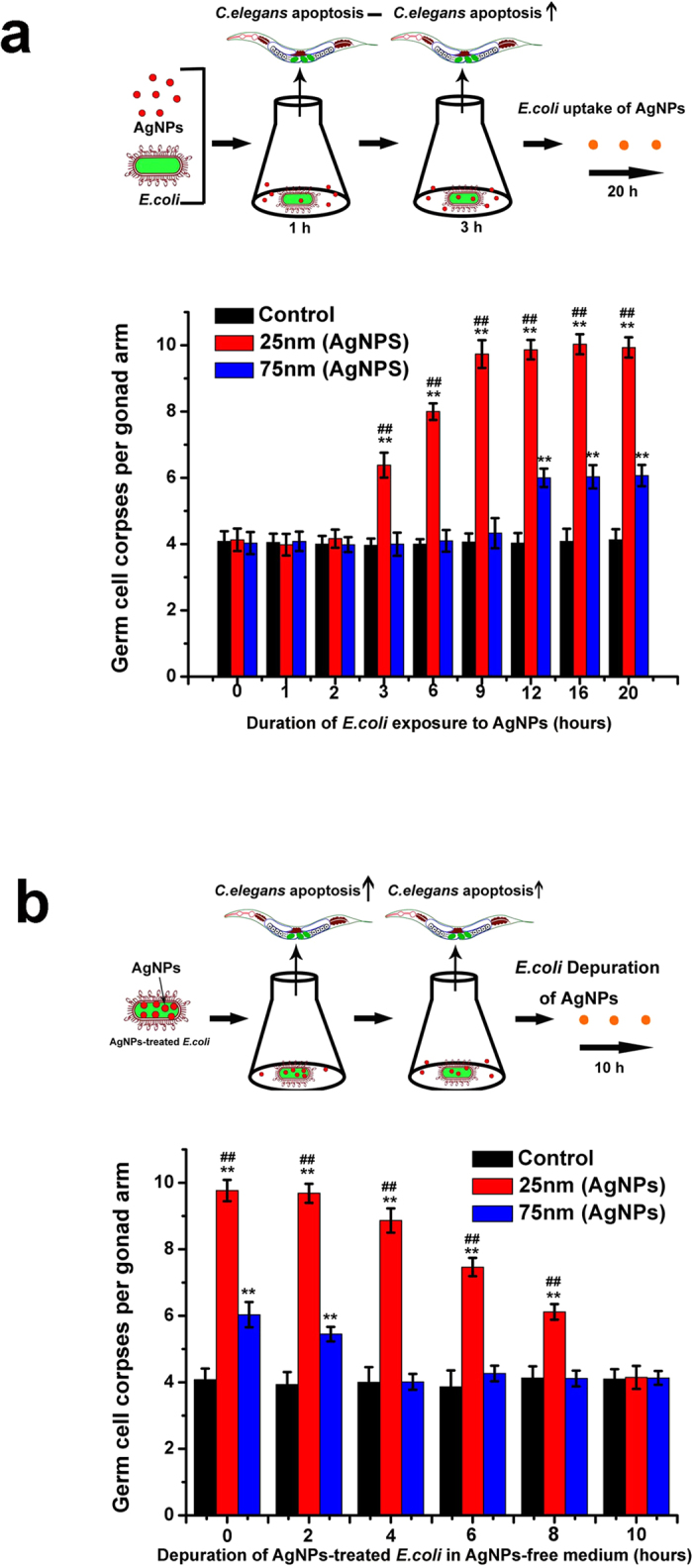
(**a**) Duration of *E. coli* exposure to AgNPs (25 μg/mL) on the induction of germ cell death in *C. elegans* through the food chain. At the indicated times, AgNPs-treated *E. coli* were collected to remove free AgNPs and were fed to *C. elegans* for 24 h. Then, the germ cell death in *C. elegans* was counted. (**b**) Depuration of AgNP-treated (25 μg/mL) *E. coli* cultured in NPs-free medium on the induction of germ cell death in *C. elegans* through the food chain. *E. coli* were cultured in LB containing 25 μg/mL AgNPs for 12 h. Then, the *E. coli* were collected to remove the AgNPs and re-cultured in AgNPs-free LB medium for the indicated times. Next, the *E. coli* were collected and fed to *C. elegans* for 24 h. Then, the germ cell death in *C. elegans* was counted. Significant difference (**p < 0.01) from control, significant difference (^##^*p* < 0.01) between 25 nm and 75 nm AgNPs-treated groups.

We have thus further demonstrated that AgNPs could induce germ cell death in *C. elegans* through the food chain. We hypothesized that this effect was dependent on the accumulation of AgNPs by *E. coli*. To confirm our hypothesis, we first exposed *E. coli* to AgNPs for different times before feeding to *C. elegans*. As expected, AgNPs-treated *E. coli* of early exposure times (≤2 h) had no germ cell death effect for either size of the nanoparticles. *E. coli* cells that had been exposed to AgNPs for 3 h resulted in a significant increase in germ cell death, but only for the 25 nm AgNPs. In the case of 75 nm AgNPs treated group, there was no significant difference in germ cell death relative to the control group up to the exposure time of 12 h (Fig. 3a). However, the toxicity of the two types of AgNPs in *C. elegans* reached a plateau after 12 h, indicating that the amount of AgNPs accumulated in *E. coli* had achieved saturation. After exposure to the AgNPs for 12 h, the *E. coli* cells were separated from the nanoparticles and allowed a clearance period (0–10 h) before being fed to *C. elegans*. There was a depuration-dependent decrease in AgNP toxicity to germ cells, where the 75 nm AgNPs did not induce germ cell death increase after a clearance period of 4 h, whereas the 25 nm AgNPs-treated group needed 8 h of depuration. No obvious increase in germ cell death was observed after 10 h of depuration for both AgNPs groups (Fig. 3b). These results indicate that the 25 nm AgNPs were taken up more easily by the *E. coli* cells, and depuration of the 25 nm AgNPs was slower than that of the 75 nm AgNPs in the food chain.

### AgNPs affect the reproduction and life cycle of *C. elegans* through the food chain

**Figure 4:**
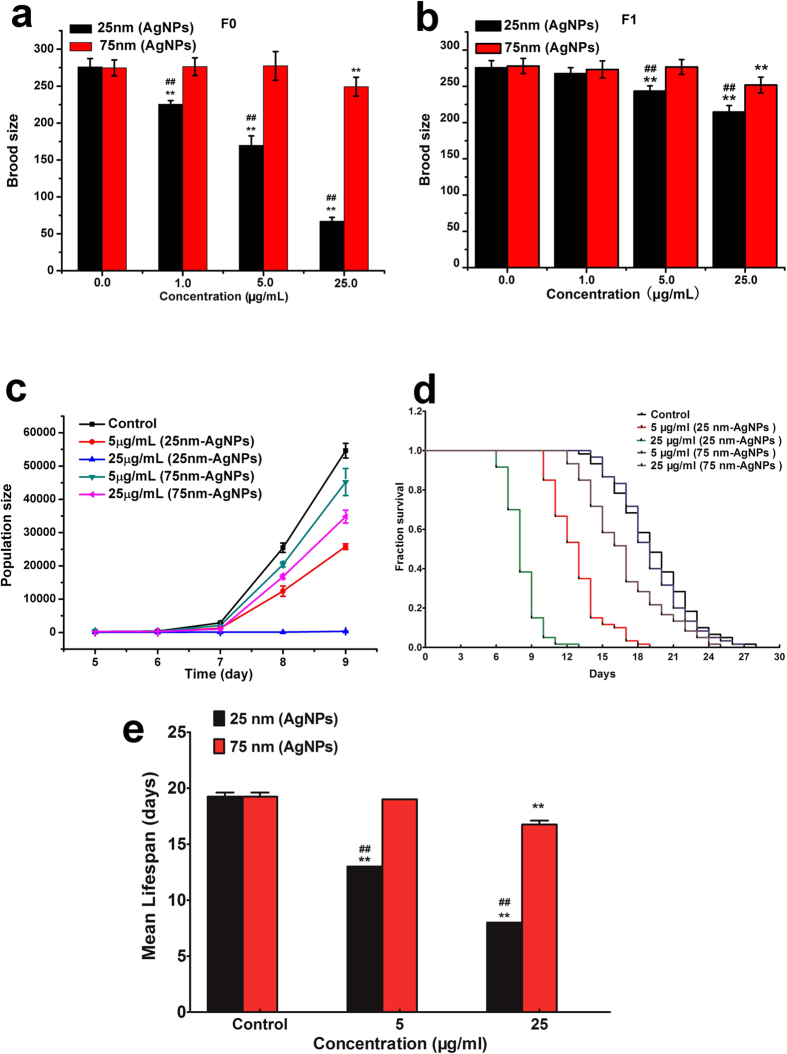
Comparison of the toxicological effects of AgNP to *C. elegans* through the food chain. (**a**) Brood size assay in F0 generation. The F0 generation was fed with AgNPs-treated *E. coli*. At least 20 *C. elegans* were used in each group, and they were transferred to new plates every 48 h until reproduction ceased. The number of larvae was counted. This assay was performed in three independent experiments. (**b**) Brood size assay in F1 generation. F1 generation was treated under AgNPs-free condition. (**c**) Population growth. The population size on days 5 to 9 of worms fed *E. coli* treated with different sized AgNPs; 20 worms were used in this assay. (**d**) Life span assays. Worms were grown on NGM plates with AgNPs-treated *E. coli*. The mortality of each group was determined by counting the numbers of live and dead animals daily. The number of *C. elegans* used in each group was 60, and three independent experiments were performed. (**e**) The mean life span of the *C. elegans* in each group was calculated from the survival curves in d. Significant difference (**p < 0.01) from control, significant difference (^##^*p* < 0.01) between 25 nm and 75 nm AgNPs-treated group.

To further confirm that AgNPs are toxic to *C. elegans* through the food chain, we also used brood size, population size and life span as endpoints. Brood size and population size provide an indication of the effects of a potential toxicant on development. As shown in Fig. 4a,b, *C. elegans* fed AgNPs-treated *E. coli* showed a decreased brood size. After removal of the offspring to the AgNPs-free condition, the brood size of the F1 generation (produced from F0 parents fed 5 and 25 μg/mL AgNPs-treated *E. coli*) was also significantly decreased compared with that of the control. Moreover, the population size, which is a consequence of both survival and all the factors that contribute to reproduction at any given time, showed a dose-dependent decrease when *C. elegans* worms were fed AgNP-treated *E. coli* (Fig. 4c). The ability of eliciting a brood size and population size decrease was in the order of 25 nm AgNPs >75 nm AgNPs, which was similar to the trend for germ cell death. These results indicate that AgNPs, with size-dependent, transferred to *C. elegans* through *E. coli* could induce a serious decrease in the reproduction rate of the parental *C. elegans* and F1 generation.

To investigate the transfer of the toxic effects of AgNPs to the lifespan of *C. elegans*, nematodes were fed *E. coli* exposed to varying concentrations of nanoparticles. As shown in Fig. 4d, size- and dose-dependent effects of the AgNPs on longevity were evident. The sharpest decrease in mean lifespan was observed in worms treated with 25 nm AgNP through the food chain compared with the control worms (8 vs. 19 days) (Fig. 4e). Our results reveal that AgNPs accumulating in species of lower trophic levels are a potential threat to higher trophic levels in the environment.

### Accumulation of AgNPs in *E. coli*

**Figure 5:**
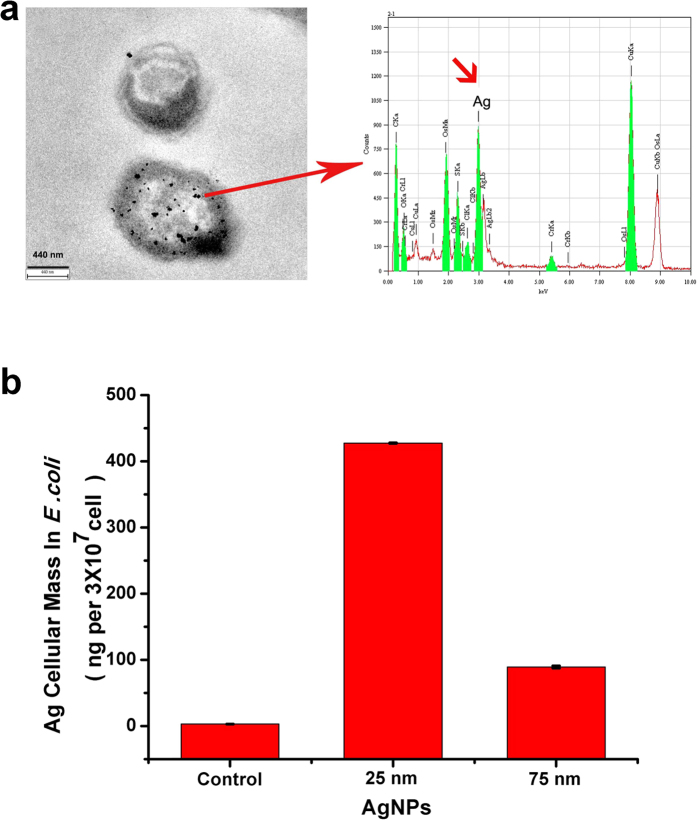
Accumulation of AgNPs in *E. coli*. (**a**) TEM analysis of the intracellular distribution of 25 nm AgNPs within *E. coli* treated with 25 μg/mL for 12 h (Left). Scale bar equals 400 nm. EDS spectra for the AgNPs in *E. coli* (Right). (**b**) Uptake of AgNPs by *E. coli*. After exposure to 25 μg/mL AgNPs for 12 h, 3 × 10^7^ *E. coli* cells were collected by flow cytometry, and the cellular dose of AgNPs in the *E. coli* was measured using ICP-AES.

The accumulation of different sizes of AgNPs in the food chain was studied to elucidate the possible mechanism of their toxicity. *E. coli* was cultivated in LB medium with AgNPs for 12 h. TEM analyses showed AgNP uptake into the *E. coli* cells (Fig. 5a). Moreover, energy-dispersive X-ray spectroscopy (EDS) clearly detected Ag in the *E. coli* cells treated with AgNPs. In addition, only small particles or aggregates of AgNPs were found in the cells. Larger aggregates were not internalized in *E. coli*. This was supported by the presence of large aggregates of AgNPs in the surrounding environment of the bacteria (Fig. S2). To further confirm and quantify the accumulation of AgNPs in *E. coli*, the total Ag content in the cells were determined by inductively coupled plasma atomic emission spectroscopy (ICP-AES). To avoid the influence of large aggregates precipitating with *E. coli* during the centrifugation washing steps, flow cytometry was used to separate the *E. coli* cells from the large AgNP aggregates. After exposure to 25 μg/mL AgNPs for 12 h, 3 × 10^7^ *E. coli* cells were collected, and the cellular dose of AgNPs in the *E. coli* was found to be 14.25 and 2.95 pg per 10^3^ cells for the 25 nm and 75 nm AgNPs, respectively (Fig. 5b, Table S1). However, in the AgNO_3_ exposure control (2.5 μg/mL), ICP-AES did not detect any Ag content in the *E. coli* cells (data not shown). The results demonstrate that bacteria accumulate AgNPs from the surrounding medium, leading to a total cellular Ag content that is significantly higher than that in the medium with a volumetric concentration factor^13^ of 877 and 182 for 25 nm and 75 nm AgNPs, respectively (Table S1). These data also confirm that the small AgNPs are accumulated more easily than the larger nanoparticles in *E. coli*.

### Imaging and quantitation of AgNPs in *C. elegans* through the food chain

**Figure 6:**
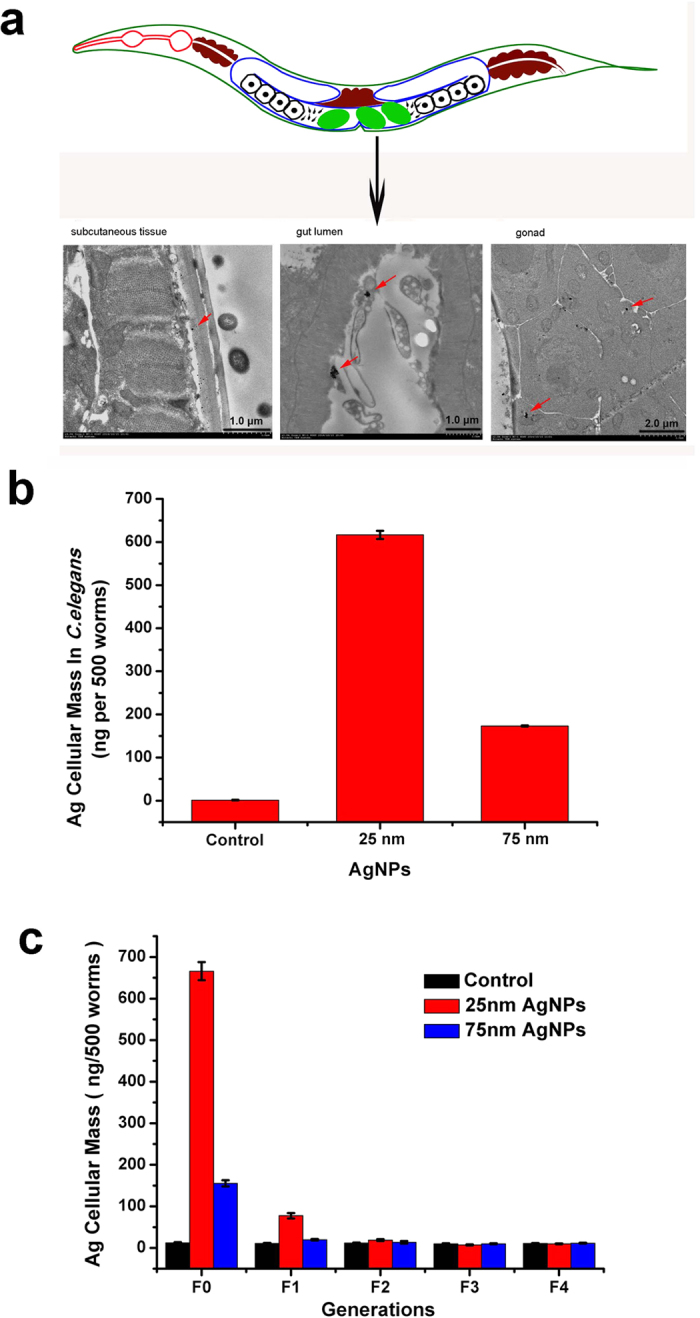
Uptake and accumulation of AgNPs in *C. elegans* through the food chain. (**a**) TEM images of intracellular localization of AgNPs in *C. elegans* fed AgNPs-treated *E. coli* included the subcutaneous tissue, gut lumen, and gonad. The red arrows indicate AgNPs. (**b**) Accumulation of AgNPs in *C. elegans* through the food chain. After being fed AgNP-treated *E. coli* for 10 d, 500 worms were selected, and the cellular dose of AgNPs in *C. elegans* was measured using ICP-AES. (**c**) AgNPs that had accumulated in *C. elegans* through the food chain were passed down from parent to the offspring. Data are shown for the F0 generation fed AgNP-treated *E. coli* and generations F1 to F4 fed *E. coli* not treated with AgNPs. On average, more than 500 *C. elegans* were collected from generations F0 to F4, and total Ag content was determined using ICP-AES in two independent experiments. The error bars indicate SD.

Since *E. coli* could accumulate AgNPs from the aqueous medium, we next investigated whether the AgNPs could be transferred to *C. elegans* through the food chain. *C. elegans* worms at the L4 stage (L4-larvae) were fed with AgNPs-exposed *E. coli*. To detect the food uptake and distribution in specific tissues, the fed worms were first examined using darkfield hyperspectral microscopy. It was obvious that the major uptake site of the AgNPs was the intestinal tract, but some AgNPs had also entered adjacent cells, including surface epithelial cells, intestinal cells, gonad cells, and eggs (Fig. S3). We further confirmed the intracellular localization of the AgNPs using TEM coupled with EDS. The TEM images revealed that some of the small AgNPs had clearly remained in the digestive lumen, subcutaneous tissue and gonad (Fig. 6a), and EDS verified the AgNPs uptake at the intracellular level. The total Ag uptake of whole *C. elegans* organisms was also measured using ICP-AES analysis. After collecting ~500 *C. elegans* worms fed 25 μg/mL AgNP-treated *E. coli* and digesting them in strong acids, we determined the amount of Ag accumulated in the organisms to be in the range of ~1233 and 346 pg per worm for the 25 nm and 75 nm AgNPs, respectively (Fig. 6b, Table S1). We also calculated the volume-based and dry-mass-based cellular Ag concentrations, which allowed us to calculate the trophic transfer factor (TTF) (Table S1). The TTF was defined as the ratio of the Ag concentration in *C. elegans* at final trophic transfer to that in *E. coli* at initial trophic transfer. The TTF values were <1, indicating that AgNP biomagnification by the *C. elegans* had not occurred.

Notably, after removal of subsequent generations to the AgNPs-free condition, a small amount of Agwas still detected in the F1 generation that had been produced from parents exposed to 25 nm AgNP-treated *E. coli* (Fig. 6c); this may be explained by the presence of AgNPs in the eggs, found using darkfield hyperspectral microscopy (Fig. S3). This result revealed that the smaller AgNPs had bioconcentrated more easily in *C. elegans*, which was consistent with the results obtained in *E. coli*. These combined data provide strong evidence for the trophic transfer of AgNPs from *E. coli* to *C. elegans* and suggest that differences in the accumulation of the two sizes of AgNPs by *C. elegans* is a likely cause of the observed size-dependent toxicity in this nematode through the food chain.

## Discussion

Up to now, there is no doubt that AgNPs exert toxicity to bacteria and other organisms, but little is known of their potential ecotoxicity to the environment, especially of the effects of specific nanomaterial properties. In the present study, we established a simplified experimental food chain using a predator (*C. elegans*) and a bacterial prey (*E. coli*) to investigate the ecotoxicological effects of AgNPs. The exposure concentrations of AgNPs were in the range of 1–25 μg/mL, based primarily on previous studies of AgNPs as antibacterial agents^22^,^23^. Our results demonstrated that AgNPs transferred to *C. elegans* through the food chain clearly induced toxicity in the gonad cells, rendering deleterious effects on the reproduction rate and life span of the worms. More importantly, the damage caused by AgNPs could transfer to subsequent generations, affected either directly by the nanoparticles themselves or through impairment of germ cells in the parental generation, highlighting the potential genetic toxicity of AgNPs in the food chain.

The characteristics of AgNPs within the test medium are important factors influencing their toxic exposure in the environment. The dissolution and aggregation behaviors of AgNPs are dependent on surface coating. Polyvinylpyrrolidone (PVP) is an environmentally friendly polymer that stabilizes AgNPs via steric repulsion. PVP-AgNPs are very stable in the environment and are therefore less toxicity to ecological systems^24^. Our data showed that AgNP aggregation was extensive after incubation in the test medium for 12 h, but there were still some individual nano-sized particles in solution. We also found only the small-sized AgNPs could be more easily accumulated in *E. coli* (Fig. 5 and Fig. S2). There is evidence that small AgNPs can more easily penetrate gram-negative bacteria than larger AgNPs or AgNPs aggregates. For example, a study by Chamakura *et al*. using TEM combined with elemental analysis showed that AgNPs within a size range of 10–50 nm were not found in gram-negative *E. coli* after 2 h of exposure^25^. Using the same method, Yuan *et al*. suggested that AgNPs with sizes between 4 and 10 nm had the ability to gain access beyond the lipid bilayer and enter gram-negative bacteria^26^. Although these studies suggested that particle size was a contributing factor to the ability of AgNPs to enter *E. coli*, they have focused mainly on the direct interactions of AgNPs with the organisms. There is little information and only a few reports on the transport of nanoparticles through the food chain^27^. In our study, trophic transfer of AgNPs between the bacterial prey and *C. elegans* was observed. Interestingly, only small nanoparticles were found in the subcutaneous tissue, gut lumen and gonad (Fig. 6). Our results demonstrated that the small (25 nm) AgNPs easily penetrated cells of various tissues through the intestinal wall. The tissue-specific distribution of nanomaterials in *C. elegans* has been reported in some direct exposure studies^28^,^29^. We have also provided direct evidence of the trophic transfer and intracellular localization of AgNPs. More importantly, the AgNPs that had accumulated in *C. elegans* through the food chain could be transferred to the next generation (Fig. 6c), indicating that AgNP exposure through the food chain can cause toxicity to subsequent generations. However, as shown in Table S1, AgNPs biomagnification by *C. elegans* is unlikely to occur, with the TTF values being < 1. Using the blood-worm as a food chain model, Yoo-iam *et al*. have also demonstrated that the food chain transfer of AgNPs occurred only in the lower trophic groups and there was no evidence of biomagnification from food sources to consumers in their simple trophic food chain model^30^. As in our model, this may because the depuration rates of *C. elegans* are much greater, or perhaps the period of AgNPs exposure to *C. elegans* may not have been long enough for biomagnification to occur though the food chain, a fact that needs to be further clarified.

Although several studies have examined the short-term acute toxic effects of AgNPs over the past decades, work on the chronic toxicity of AgNPs within lower trophic organisms is limited. In *C. elegans*, aspects of the life cycle, including germ cell death, brood size, population growth rate, and lifespan, all vary as a consequence of food quantity and quality or conditions of environmental stress^31^. In order to explore the genotoxicity of AgNPs for *C. elegans* in the food chain, we used germ cell death, brood size, population growth rate, and lifes pan to evaluate two sizes of AgNPs. We showed that 1 μg/mL of 25 nm AgNPs, rather than 75 nm AgNPs, could cause germ cell death and reproduction inhibition and consequently affect the population growth of *C. elegans* (Figs 2 and 4). Many direct-exposure studies have reported that small AgNPs were more toxic than large particles. For example, Liu *et al*. found that 5 nm AgNPs were more toxic than 20 and 50 nm AgNPs to human cells^32^. Wang *et al*. also found that 20 nm PVP-AgNPs induced more cellular toxicity than 110 nm AgNPs^33^. Moreover, Gliga *et al*. reported that 10 nm AgNPs were much more cytotoxic than 40 and 75 nm AgNPs to human lung cells. However, there was no difference in toxicity between the 10 nm citrate- and 10 nm PVP-coated AgNPs^34^. These results indicated that the toxicity of AgNPs, not only in direct exposure but also through the food chain, was dependent on size. In addition, there was a time effect of AgNPs exposure to *E. coli* in the food chain. We found that germ cell death was induced drastically with the exposure time extended, where the 25 nm AgNPs exerted toxicity to *C. elegans* only after they had been exposed to *E. coli* for 3 h. With regard to the depuration period, the 25 nm AgNPs-treated group also needed the longest time to eliminate the toxic effects of AgNPs, which indicated that it was more difficult for the small-sized AgNPs to be excluded from the *E. coli* cell (Fig. 3). Indeed, the accumulation and depuration of nanoparticles in lower species are more variable, and are related to the surface chemistry and aggregate size of the nanoparticles^14^. These results suggest that the exposure and depuration durations should be considered as potentially important factors in the toxicity of AgNPs to *C. elegans* through the food chain.

We also investigated the generational transfer and multigenerational toxicity of AgNPs in *C. elegans*, where we found that only 25 nm AgNPs could be detected in F1 generations. The germ cell death caused by the 25 nm AgNPs was clearly increased in the F2 and F3 generations but then gradually recovered in the F4 generation. Previous reports have suggested that AgNPs could distribute to embryos and modify the embryonic development of vertebrates^35^,^36^. There was evidence that nanoparticles, which permeated the gonad of *C. elegans*, could be passed to the next generation and exert potentially toxic effects on their postembryonic development^19^,^28^. A recent study has revealed that AgNPs (10 nm) could significantly reduce the lifespan of parent nematodes (F0) as well as those of 3 subsequent generations (F1–F3)^37^. These phenomena may be due to the AgNPs that had accumulated in *C. elegans* through the food chain and the resulting transfer of impaired germ cell.

Although there is no doubt that the Ag ions can be released from AgNPs, there is no conclusive answer to date on the exact source of the AgNPs-mediated toxicity: that is whether the toxicity is due to particle-specific effects or released Ag ion effects. Previous studies have shown that Ag ions govern the toxicity of AgNPs towards bacteria^22^. Yang *et al*. reported that AgNPs toxicity was dependent on released Ag ions and AgNPs with higher Ag release were more toxic to *C. elegans*^38^. In our experiment, the amount of Ag released into solution after 12 h incubation of AgNPs in the test medium was 1 μg/mL, which is 4% of that released from 25 μg/mL AgNPs added (Fig. S4a). In order to investigate whether the released Ag ionic species could cause toxicity in the food chain, the *E. coli* was exposed to 2.5 μg/mL AgNO_3_ (representing 10% of Ag released from 25 μg/mL of AgNPs) and then fed to *C. elegans*. There was no significant effect on the germ cell death of *C. elegans* through the food chain (Fig. S4b), suggesting that the toxic effects were not related to Ag ion release. We also observed that large aggregates of AgNPs that had formed in the LB medium would coprecipitate during *E. coli* collection. These large aggregates may be toxic to *C. elegans* through direct interactions. However, we found that germ cell death was not significantly different between large-aggregate-treated *C. elegans* and the controls (Fig. S4c). Therefore, we confirmed that all toxic effects towards *C. elegans* were directly due to the AgNPs transferred by *E. coli* through the food chain. Overall, our results clearly showed that AgNPs could sizd-dependently be accumulated though the food chain and cause toxicity to high trophic levels.

## Methods

### Preparation and characterization of AgNPs

Two different sized AgNPs were investigated in our study. The 25 and 75 nm PVP BioPure^TM^ AgNPs were obtained from NanoComposix, Inc. (San Diego, CA, USA) in the form of stock dispersions (5 mg/mL, PVP coated). The morphology of the AgNPs was observed using transmission electron microscopy (TEM, JEM-2011, Japan). The hydrodynamic diameter and zeta potential of the AgNPs were characterized using a Zetasizer (Malvern Nano series, Malvern, UK).

### *C. elegans* culture conditions

Wild-type N2 *C. elegans* worms maintained in our laboratory were obtained from the Caenorhabditis Genetics Center (CGC, University of Minnesota, USA) and were maintained at 20 °C on a standard nematode growth medium (NGM) with living *Escherichia coli* OP50 bacteria as a food source. All manipulations of *C. elegans* were carried out according to the standard procedures.

### *E. coli* exposed to AgNPs and fed to *C. elegans* in the food chain experiment

To investigate the uptake and transfer of AgNPs in a food chain, we examined a simplified invertebrate food chain using *C. elegans*, and *E. coli* strain OP50 was chosen as the food source. First, *E. coli* OP50 was cultured in 5 ml of LB medium overnight (37 °C, 200 rpm). Then, 200 μL of the *E. coli* that had been cultured overnight was added to 10 mL of LB with different concentrations of AgNPs (0, 1, 5, 25 μg/mL) and was incubated at 37 °C for 12 h at 200 rpm. After being exposed for 12 h, treated *E. coli* were collected at 5000 rpm for 5 min at 4 °C and were then washed 3 times with deionized water. The final pellets were resuspended into 1 mL of deionized water. Then, an aliquot (100 μL) of the collected *E. coli* was dropped onto the surface of NGM plates (35 mm dishes). The plates were dried at 20 °C to evaporate the moisture from the NGM medium. Then, worms were placed onto NGM plates seeded with treated *E. coli* and were incubated at 20 °C until the toxicity assays were performed.

### Germ cell death, brood size, population size and lifespan assays for the assessment of AgNP toxicity Germ cell death assay

Germ cell death was measured using acridine orange (AO, Sigma)^39^. L4 larvae (50–60) were fed AgNP-treated *E. coli* for 24 h. Then, the treated worms were strained into 500 μL of AO (25 μg/mL) for 1 h in the dark at 20 °C. They were then transferred to a new NGM plate and allowed to recover for 40 min on bacterial lawns. Dead cells stained positive for AO were counted using an Olympus IX71 fluorescence microscope (Olympus, Japan).

### Brood size assay

The procedures for brood size assay were conducted as described previously^40^. Wild-type L1 larvae were fed AgNP-treated *E. coli* and were subsequently transferred onto a new plate with treated *E. coli* every day. The number of newly hatched larvae was counted. This procedure was repeated until the mother worms stopped laying eggs. The brood size was calculated by combining the numbers of hatched larvae.

### Population size assay

Population growth was measured according to a method described in a previous report^31^. In brief, 20 randomly selected worms were individually placed on an NGM plate seeded with AgNP-treated *E. coli*. The population that grew from these individual worms was assessed from 5 to 9 days.

### Life span assay

The same AgNP-treated *E. coli* feeding procedures were followed for the life span assay. Every day, worms were transferred to a new AgNP-treated *E. coli* plate and until it could be confirmed that they were dead, i.e., when they would not respond to being tapped with a pick.

### Observation of AgNP distribution in the food chain

*C. elegans* with AgNPs were imaged using hyperspectral imagery with enhanced darkfield microscopy (CytoViva, Inc.) as previously described^41^,^42^. A high-resolution darkfield condenser was directly positioned on a glass slide upon the work stage with an immersion solution and was focused until a donut ring-type light pattern was obtained. All the images were captured using a CCD camera (Olympus BX51, Japan).

AgNP distribution in the *E. coli* and *C. elegans* was also confirmed using TEM equipped with energy dispersive spectroscopy (EDS). After being exposed to AgNPs, *E. coli* cells and *C. elegans* were collected and fixed with 2.5% glutaraldehyde. Then, the samples was washed 3 times with 0.1 M phosphate buffer and postfixed with 1% osmium tetroxide in phosphate buffer. The fixed samples were then dehydrated in ethanol and embedded in Epon 812 resin. The resin specimens were sectioned using an ultramicrotome with a diamond knife. The thin sections were observed using TEM equipped with EDS.

### Calculations of the concentration of the total Ag used in the food chain experiment

To avoid the influence of large aggregates precipitating with *E. coli* during the centrifugation washing steps, flow cytometry was used to separate the *E. coli* from the AgNP suspensions. *E. coli* OP50 expressing a GFP (OP50-GFP, from CGC) were used in the AgNP treatment. After exposure, the OP50-GFP cells were washed 3 times with deionized water. The final pellets were resuspended in deionized water. The flow cytometry procedure was modified according to a previous description^43^. In brief, a laser output power of 0.5 W was used for measuring forward scatter (FSC), side scatter (SSC) and green fluorescence intensity (FL1). For sorting out the OP50-GFP cells, a 488 nm argon laser was used for excitation. The drop drive frequency was set to ~26 kHz, and the system threshold rate was kept under 8000 s^−1^. Finally, 3 × 10^7^ *E. coli* were collected for each sample. The collected samples were acidified with 10% HNO_3_ and allowed to digest at 37 °C overnight prior to the quantification of silver concentration by inductively coupled plasma mass spectrometry (ICP-AES, PlasmaQuad3, VG Elemental). For quantitation of the total Ag in the *C. elegans*, the worms were fed AgNP-treated *E. coli* OP50. After the indicated times, 500 worms were selected and analyzed using ICP-MS.

The method used for the calculation of the trophic transfer factor (TTF) was modified according to a previous study^13^. The total masses of Ag out of the mass of the counted organisms, the *E. coli* and worms, were measured using ICP-AES. Ag mass per cell or worm was determined by dividing these values by the organism count in each sample. Volume-based cellular Ag concentrations were then calculated by dividing the Ag mass per organism by the organism volume. The volumes of the *E. coli* and worms were 0.65 μm^3^ and 5 × 10^6^ μm^3^, respectively, as reported in the literature^44^,^45^. *E. coli* dry mass (0.71 pg) was calculated by multiplying the cell volume (0.65 μm^3^) by cell density (1.09 mg/μL)^46^. For the worms, a cellular dry mass of 5 μg was estimated from 1000 worms using a microbalance. TTF was determined as the worm: *E. coli* ratios of cellular Ag concentrations on a volume basis and on a dry mass basis, where cellular Ag concentration of *C. elegans* was Ag in *C. elegans* at final trophic transfer experiment (*C.ele* 5d in Table S1 indicated Ag concentration in worms which fed with AgNPs-treated *E. coli* for 5 days), and cellular Ag concentration of *E. coli* was Ag in *E. coli* at initial trophic transfer experiment (*E. coli* exposed to AgNPs for 12 h and fed to *C. elegans* to start trophic transfer experiment was defined *E. coli* 0 h in Table S1). The two TFFs (volume and dry mass based) for each treatment differed by less than 10%, and an average was obtained.

### Statistical analysis

All the results are expressed as the mean ± SD. One-way analysis of variance (ANOVA) was used to analyze the mean differences among groups compared with the control groups, and two-way ANOVA was used to compare the mean differences between groups. The criterion for statistical significance was *p* < 0.05.

## Additional Information

**How to cite this article**: Luo, X. *et al*. Insights into the Ecotoxicity of Silver Nanoparticles Transferred from *Escherichia coli* to *Caenorhabditis elegans*. *Sci. Rep*. **6**, 36465; doi: 10.1038/srep36465 (2016).

**Publisher’s note:** Springer Nature remains neutral with regard to jurisdictional claims in published maps and institutional affiliations.

## Supplementary Material

Supplementary Information

## References

[b1] Ahamed M., Alsalhi M. S. & Siddiqui M. K. Silver nanoparticle applications and human health. Clin. Chim. Acta 411, 1841–1848 (2010). 20719239 10.1016/j.cca.2010.08.016

[b2] Sintubin L., Verstraete W. & Boon N. Biologically produced nanosilver: current state and future perspectives. Biotechnol. Bioeng. 109, 2422–2436 (2012). 22674445 10.1002/bit.24570

[b3] Seltenrich N. Nanosilver: weighing the risks and benefits. Environ. Health Perspect. 121, A220–A225 (2013). 23816826 10.1289/ehp.121-a220 PMC3702006

[b4] Maurer-Jones M. A., Gunsolus I. L., Murphy C. J. & Haynes C. L. Toxicity of engineered nanoparticles in the environment. Anal. Chem. 85, 3036–3049 (2013). 23427995 10.1021/ac303636s PMC4104669

[b5] Wijnhoven S. W. P. . Nano-silver - a review of available data and knowledge gaps in human and environmental risk assessment. Nanotoxicology 3, 109–138 (2009).

[b6] Massarsky A., Trudeau V. L. & Moon T. W. Predicting the environmental impact of nanosilver. Environ. Toxicol. Pharmacol. 38, 861–873 (2014). 25461546 10.1016/j.etap.2014.10.006

[b7] Li W. R. . Antibacterial activity and mechanism of silver nanoparticles on *Escherichia coli*. Appl. Microbiol. Biotechnol. 85, 1115–1122 (2010). 19669753 10.1007/s00253-009-2159-5

[b8] Rahman M. F. . Expression of genes related to oxidative stress in the mouse brain after exposure to silver-25 nanoparticles. Toxicol. Lett. 187, 15–21 (2009). 19429238 10.1016/j.toxlet.2009.01.020

[b9] Asharani P. V., Hande M. P. & Valiyaveettil S. Anti-proliferative activity of silver nanoparticles. BMC Cell Biol. 10, 65 (2009). 19761582 10.1186/1471-2121-10-65 PMC2759918

[b10] Roh J. Y. . Ecotoxicity of Silver Nanoparticles on the Soil Nematode Caenorhabditis elegans Using Functional Ecotoxicogenomics. Environ.Sci. Technol. 43, 3933–3940 (2009). 19544910 10.1021/es803477u

[b11] Park S. Y. . Ecotoxicity of bare and coated silver nanoparticles in the aquatic midge, Chironomus riparius. Environ. Toxicol. Chem. 34, 2023–2032 (2015). 25892495 10.1002/etc.3019

[b12] Hund-Rinke K. . Regulatory Ecotoxicity Testing of Nanomaterials - Proposed Modifications of OECD Test Guidelines Based on Laboratory Experience with Silver and Titanium Dioxide nanoparticles. Nanotoxicology. 1–16 (2016). 10.1080/17435390.2016.1229517 27592624

[b13] Werlin R. . Biomagnification of cadmium selenide quantum dots in a simple experimental microbial food chain. Nat. Nanotechnol. 6, 65–71 (2011). 21170041 10.1038/nnano.2010.251

[b14] Holbrook R. D., Murphy K. E., Morrow J. B. & Cole K. D. Trophic transfer of nanoparticles in a simplified invertebrate food web. Nat. Nanotechnol. 3, 352–355 (2008). 18654546 10.1038/nnano.2008.110

[b15] Judy J. D., Unrine J. M. & Bertsch P. M. Evidence for biomagnification of gold nanoparticles within a terrestrial food chain. Environ. Sci. Technol. 45, 776–781 (2011). 21128683 10.1021/es103031a

[b16] McTeer J., Dean A. P., White K. N. & Pittman J. K. Bioaccumulation of silver nanoparticles into Daphnia magna from a freshwater algal diet and the impact of phosphate availability. Nanotoxicology 8, 305–316 (2014). 23421707 10.3109/17435390.2013.778346

[b17] Chen J., Li H., Han X. & Wei X. Transmission and Accumulation of Nano-TiO2 in a 2-Step Food Chain (Scenedesmus obliquus to Daphnia magna). Bull Environ. Contam. Toxicol. 95, 145–149 (2015). 26091814 10.1007/s00128-015-1580-y

[b18] Chae Y. & An Y. J. Toxicity and transfer of polyvinylpyrrolidone-coated silver nanowires in an aquatic food chain consisting of algae, water fleas, and zebrafish. Aquat. Toxicol. 173, 94–104 (2016). 26854872 10.1016/j.aquatox.2016.01.011

[b19] Kim S. W., Kwak J. I. & An Y. J. Multigenerational study of gold nanoparticles in Caenorhabditis elegans: transgenerational effect of maternal exposure. Environ. Sci. Technol. 47, 5393–5399 (2013). 23590387 10.1021/es304511z

[b20] Guo X. . Synergistic effects induced by a low dose of diesel particulate extract and ultraviolet-A in Caenorhabditis elegans: DNA damage-triggered germ cell apoptosis. Chem. Res. Toxicol. 27, 990–1001 (2014). 24841043 10.1021/tx500137f PMC4067152

[b21] Du H. . Endosulfan isomers and sulfate metabolite induced reproductive toxicity in Caenorhabditis elegans involves genotoxic response genes. Environ. Sci. Technol. 49, 2460–2468 (2015). 25612189 10.1021/es504837z

[b22] Xiu Z. M., Zhang Q. B., Puppala H. L., Colvin V. L. & Alvarez P. J. J. Negligible Particle-Specific Antibacterial Activity of Silver Nanoparticles. Nano Lett. 12, 4271–4275 (2012). 22765771 10.1021/nl301934w

[b23] Chernousova S. & Epple M. Silver as Antibacterial Agent: Ion, Nanoparticle, and Metal. Angew. Chem. Int. Edit 52, 1636–1653 (2013). 10.1002/anie.201205923 23255416

[b24] Li Y., Zhang W., Niu J. & Chen Y. Surface-coating-dependent dissolution, aggregation, and reactive oxygen species (ROS) generation of silver nanoparticles under different irradiation conditions. Environ. Sci. Technol. 47, 10293–10301 (2013). 23952964 10.1021/es400945v

[b25] Chamakura K., Perez-Ballestero R., Luo Z., Bashir S. & Liu J. Comparison of bactericidal activities of silver nanoparticles with common chemical disinfectants. Colloids Surf. B Biointerfaces 84, 88–96 (2011). 21227664 10.1016/j.colsurfb.2010.12.020

[b26] Yuan Z. . Interaction of silver nanoparticles with pure nitrifying bacteria. Chemosphere 90, 1404–1411 (2013). 22985593 10.1016/j.chemosphere.2012.08.032

[b27] Hou W. C., Westerhoff P. & Posner J. D. Biological accumulation of engineered nanomaterials: a review of current knowledge. Environ. Sci. Process Impacts 15, 103–122 (2013). 24592431 10.1039/c2em30686g

[b28] Mohan N., Chen C. S., Hsieh H. H., Wu Y. C. & Chang H. C. *In vivo* imaging and toxicity assessments of fluorescent nanodiamonds in Caenorhabditis elegans. Nano Lett. 10, 3692–3699 (2010). 20677785 10.1021/nl1021909

[b29] Qu Y. . Full assessment of fate and physiological behavior of quantum dots utilizing Caenorhabditis elegans as a model organism. Nano Lett. 11, 3174–3183 (2011). 21721562 10.1021/nl201391e

[b30] Yoo-iam M., Chaichana R. & Satapanajaru T. Toxicity, bioaccumulation and biomagnification of silver nanoparticles in green algae (Chlorella sp.), water flea (Moina macrocopa), blood worm (Chironomus spp.) and silver barb (Barbonymus gonionotus). Chem. Spec. Bioavailab. 26, 257–265 (2014).

[b31] Harvey S. C., Shorto A. & Viney M. E. Quantitative genetic analysis of life-history traits of Caenorhabditis elegans in stressful environments. BMC Evol. Biol. 8, 15 (2008). 18211672 10.1186/1471-2148-8-15 PMC2267162

[b32] Liu W. . Impact of silver nanoparticles on human cells: effect of particle size. Nanotoxicology 4, 319–330 (2010). 20795913 10.3109/17435390.2010.483745

[b33] Wang X. . Use of Coated Silver Nanoparticles to Understand the Relationship of Particle Dissolution and Bioavailability to Cell and Lung Toxicological Potential. Small 10, 385–398 (2014). 24039004 10.1002/smll.201301597 PMC4001734

[b34] Gliga A. R., Skoglund S., Wallinder I. O., Fadeel B. & Karlsson H. L. Size-dependent cytotoxicity of silver nanoparticles in human lung cells: the role of cellular uptake, agglomeration and Ag release. Part. Fibre Toxicol. 11, 11 (2014). 24529161 10.1186/1743-8977-11-11 PMC3933429

[b35] Ahamed M. . DNA damage response to different surface chemistry of silver nanoparticles in mammalian cells. Toxicol. Appl. Pharmacol. 233, 404–410 (2008). 18930072 10.1016/j.taap.2008.09.015

[b36] Austin C. A. . Distribution of silver nanoparticles in pregnant mice and developing embryos. Nanotoxicology 6, 912–922 (2012). 22023110 10.3109/17435390.2011.626539

[b37] Contreras E. Q., Puppala H. L., Escalera G., Zhong W. & Colvin V. L. Size-dependent impacts of silver nanoparticles on the lifespan, fertility, growth, and locomotion of Caenorhabditis elegans. Environ. Toxicol. Chem. 33, 2716–2723 (2014). 25088842 10.1002/etc.2705 PMC4331122

[b38] Yang X. Y. . Mechanism of Silver Nanoparticle Toxicity Is Dependent on Dissolved Silver and Surface Coating in Caenorhabditis elegans. Environ. Sci. Technol. 46, 1119–1127 (2012). 22148238 10.1021/es202417t

[b39] Kelly K. O., Dernburg A. F., Stanfield G. M. & Villeneuve A. M. Caenorhabditis elegans msh-5 is required for both normal and radiation-induced meiotic crossing over but not for completion of meiosis. Genetics 156, 617–630 (2000). 11014811 10.1093/genetics/156.2.617 PMC1461284

[b40] Craig A. L., Moser S. C., Bailly A. P. & Gartner A. Methods for studying the DNA damage response in the Caenorhabdatis elegans germ line. Methods Cell Biol. 107, 321–352 (2012). 22226529 10.1016/B978-0-12-394620-1.00011-4

[b41] Badireddy A. R., Wiesner M. R. & Liu J. Detection, characterization, and abundance of engineered nanoparticles in complex waters by hyperspectral imagery with enhanced Darkfield microscopy. Environ. Sci. Technol. 46, 10081–10088 (2012). 22906208 10.1021/es204140s

[b42] Yang X. . Silver nanoparticle behavior, uptake, and toxicity in Caenorhabditis elegans: effects of natural organic matter. Environ. Sci. Technol. 48, 3486–3495 (2014). 24568198 10.1021/es404444n

[b43] Uchiyama T. & Watanabe K. Substrate-induced gene expression (SIGEX) screening of metagenome libraries. Nat Protoc. 3, 1202–1212 (2008). 18600226 10.1038/nprot.2008.96

[b44] Kubitschek H. E. Cell volume increase in *Escherichia coli* after shifts to richer media. J. Bacteriol. 172, 94–101 (1990). 2403552 10.1128/jb.172.1.94-101.1990 PMC208405

[b45] McCulloch D. & Gems D. Body size, insulin/IGF signaling and aging in the nematode Caenorhabditis elegans. Exp. Gerontol. 38, 129–136 (2003). 12543270 10.1016/s0531-5565(02)00147-x

[b46] Kubitschek H. E., Baldwin W. W., Schroeter S. J. & Graetzer R. Independence of buoyant cell density and growth rate in *Escherichia coli*. J. Bacteriol. 158, 296–299 (1984). 6370960 10.1128/jb.158.1.296-299.1984 PMC215411

