# Supplementary material for: Insights into the Ecotoxicity of Silver Nanoparticles Transferred from Escherichia coli to Caenorhabditis elegans
Source: Sci Rep. 2016 Nov 4;6:36465. doi: 10.1038/srep36465 (PMC5095556; doi:10.1038/srep36465)
Supplement: Supplementary Information [file srep36465-s1.pdf]

**Supplementary Information for**

# Insights into the Ecotoxicity of Silver Nanoparticles Transferred from *Escherichia coli* to *Caenorhabditis elegans*

Xun Luo[1,2,4], Shengmin Xu[1,3*],Yaning Yang[1,2], Luzhi Li[1,3], Shaopeng Chen[1,3], An Xu[1,3], and Lijun Wu[1,2,3*]

[1]Key Laboratory of Ion Beam Bioengineering, Hefei Institutes of Physical Science, Chinese Academy of Sciences, Hefei, Anhui 230031, China

[2]School of Life Sciences, University of Science and Technology of China, Hefei 230026, Anhui, China

[3]Key Laboratory of Environmental Toxicology and Pollution Control Technology of Anhui Province, Hefei, Anhui 230031, China

[4]School of Bioengineering, Huainan Normal University, Huainan 232038, China

[*]To whom correspondence should be addressed at Key Laboratory of Ion Beam Bioengineering, Hefei Institutes of Physical Science, Chinese Academy of Sciences, P. O. Box 1138, Hefei, Anhui 230031, China. Tel: 86-551-65591602; Fax: 86-551-65595670. Email: shmxu@mail.ustc.edu.cn (S.M.X.) & ljw@ipp.ac.cn (L.J.W.)

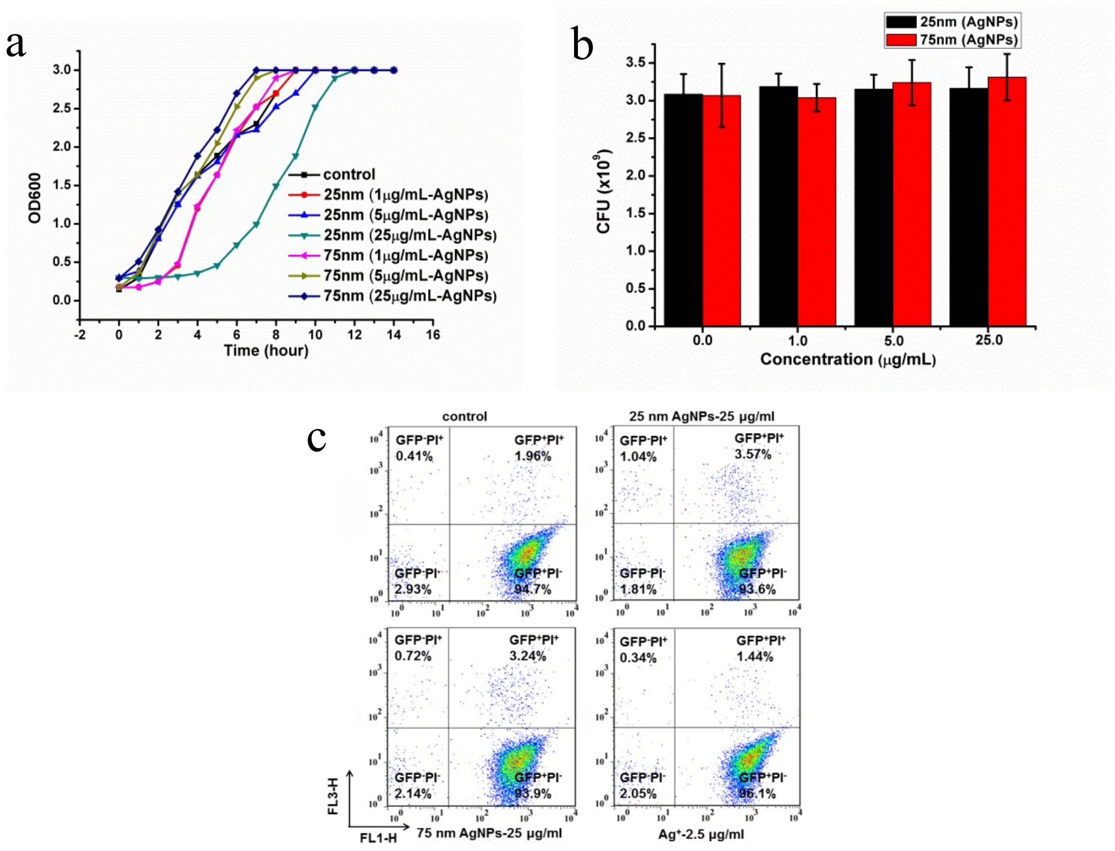


**Percentage of live *E. coli* exposed to AgNPs or $Ag^+$ for 12 h**

| Group | Control | 25 nm AgNPs | 75 nm AgNPs | $Ag^+$ |
|---|---|---|---|---|
| Alive (%) | 94.7 | 93.6 | 93.9 | 96.1 |

**Figure S1. The toxicity of AgNPs in *E. coli*.** **(a)** Growth curves of AgNPs-treated *E. coli* were determined by OD600 for up to 14 h. The cell densities were unaltered by the presence of AgNPs except for 25 μg/mL of 25 nm AgNPs. **(b)** Colony formation was determined at 12 h. No differences were observed at 12 h with the presence of any AgNPs size. Error bars indicate SD (n=3). **(c)** Dot plot of *E. coli* treated with AgNPs and silver ions. *E. coli* OP50-GFP was treated with AgNPs and silver ions at indicated dose for 12 h. After that, *E. coli* was collected and washed 3 times with PBS. PI was added to the suspensions and incubated for 30 min in the dark. Subsequently, the fluorescence of the cells was measured by flow cytometer (BD Calibur, BD biosciences). GFP-positive and PI-negative (GFP+PI-) bacteria in the lower right quadrant was alive.

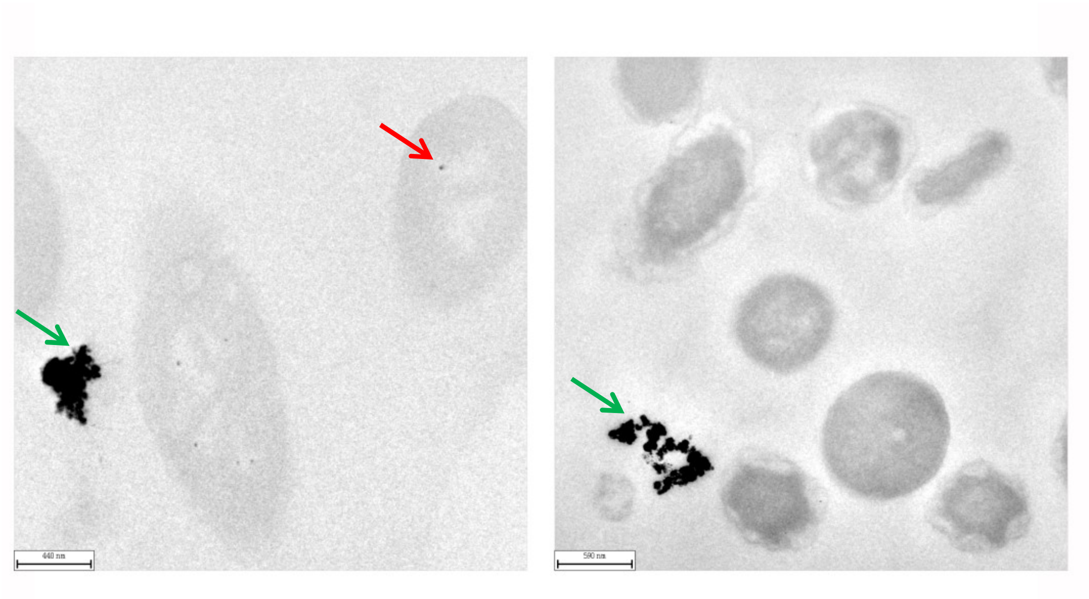

**Figure S2 Distribution of 25 nm AgNPs inside and outside the *E. coli*.** Small AgNPs were always located within the cells, and large aggregates were not found in the cells. The red arrows indicate small AgNPs, and the green arrows indicate large aggregates. Scale bars equal 440 nm (left) and 590 nm (right).

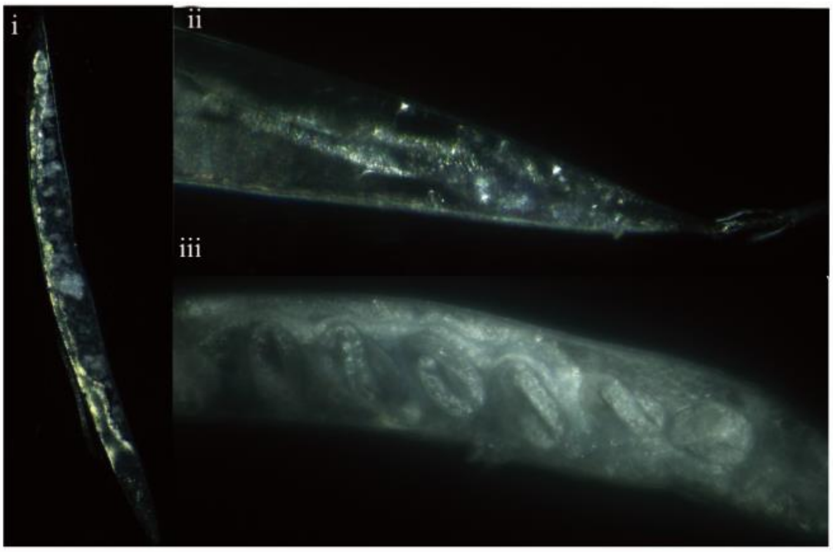


**Figure S3. Representative darkfield images of 25 nm AgNP distribution in *C. elegans* transferred from *E. coli*.** (i) Whole-body darkfield image indicating that AgNPs were mostly distributed in the gut. (ii) Epithelium and muscle. (iii) Gonad and eggs. The observation of AgNPs in (ii) and (iii) indicated that the AgNPs had entered the adjacent cells and eggs from the gut.

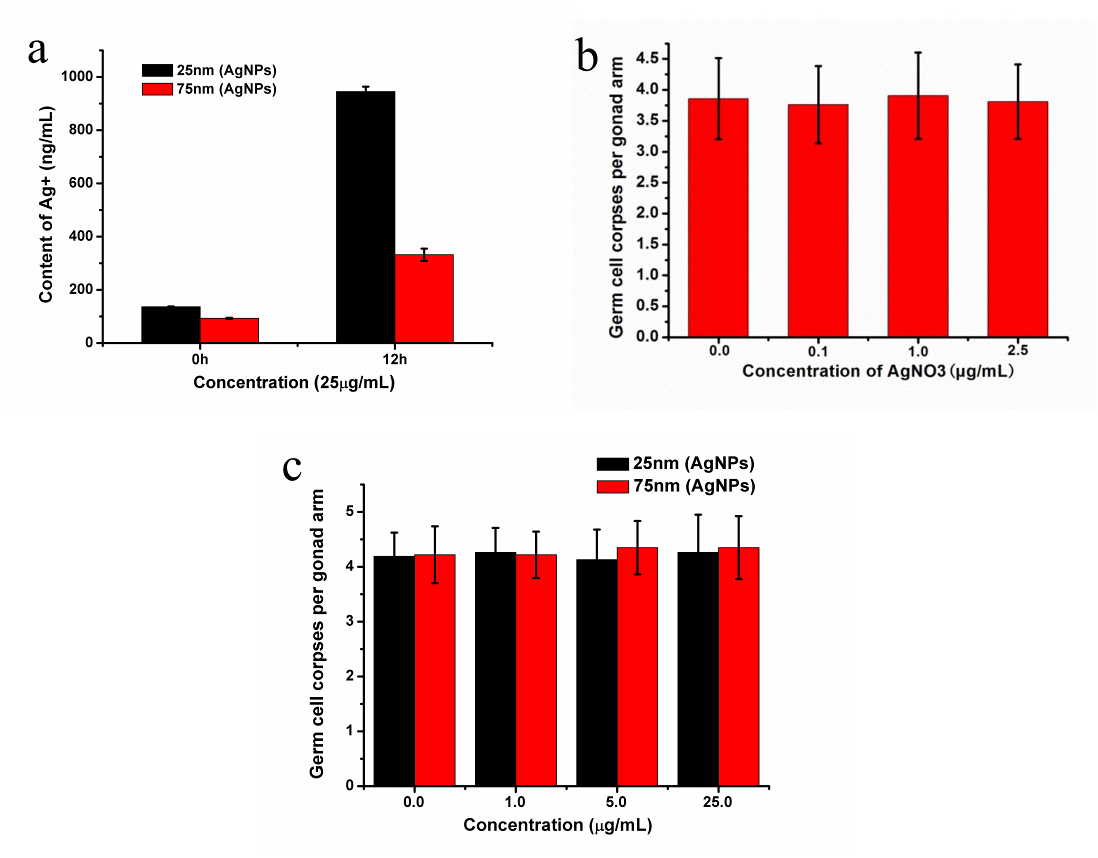


**Figure S4 (a)** Quantification of the Ag release in the LB medium. The amount of Ag released in the LB medium after 12 was measured using ICP-MS. The 25 nm AgNPs showed the highest Ag release in the LB medium (~4%) from the total number of added AgNPs (25 μg/mL). The data represent the means of duplicate experiments with SD. **(b)** Germ cell death in *C. elegans* induced by exposure to Ag ions through the food chain. The highest dose of Ag ions (2.5 μg/mL, 10% of that released from 25 μg/mL of AgNPs) exerted no effects on the induction of germ cell death. **(c)** Large aggregates of AgNPs formed in LB could not induce germ cell death in *C. elegans*. AgNPs were cultured with *E. coli* in LB medium for 12 h. After being centrifuged at 4500 rpm for 15 min, the pellet of larger AgNPs was fed to the *C. elegans* with the *E. coli*.

**Table S1 Cellular Ag mass and concentration measurements in the food chain experiment with calculated trophic transfer factors.**

| 1 | 2. Ag Cellular Mass (pg/cell*$10^3$) | 3. Volume-based Ag Cellular Concentration (mg/L) | 4. TTF Volume Basis | 5. Dry-mass-based Ag Cellular Concentration (μg/g) | 6. TTF Mass Basis | 7. Avg. TTF |
|---|---|---|---|---|---|---|
| 25 nm AgNPs treatment | | | | | | |
| *E. coli* (0 h) | 14.25±0.07 | 21,923±109 | 0.011 | 20,070±100 | 0.012 | 0.012 |
| *C. ele* (5 d) | 1,233,000±27,068 | 247±5.37 | | 247±5.37 | | |
| 75 nm AgNPs treatment | | | | | | |
| *E. coli* (0 h) | 2.95±0.07 | 4,538±109 | 0.015 | 4,155±100 | 0.017 | 0.016 |
| *C. ele* (5 d) | 346,400±3281 | 69±0.64 | | 69±0.63 | | |
